# Oat polar lipids and sunflower lecithin similarly improve cardiometabolic risk markers and appetite controlling hormone responses after breakfast and a subsequent lunch. A randomized crossover study in healthy adults

**DOI:** 10.3389/fnut.2024.1497844

**Published:** 2024-11-06

**Authors:** Mohammad Mukul Hossain, Juscelino Tovar, Lieselotte Cloetens, Soraya de Kam, Anne Nilsson

**Affiliations:** ^1^Division of Food and Pharma, Lund University, Lund, Sweden; ^2^Division of Pure and Applied Biochemistry, Lund University, Lund, Sweden

**Keywords:** oat polar lipids, glycaemia, GLP-1, PYY, lecithin

## Abstract

**Introduction:**

The alarming global increase in lifestyle-related disorders such as obesity and type 2 diabetes mellitus (T2DM) has increased during the last several decades. Poor dietary choices significantly contribute to this increase and prevention measures are urgently needed. Dietary intake of bioactive compounds found in foods are linked to a decrease likelihood of these disorders. For this purpose, a randomized crossover meal study was performed to compare the postprandial metabolic effects of lecithin and oat polar lipids in healthy subjects.

**Materials and methods:**

Eighteen young healthy subjects ingested test meals enriched with lecithin, oat polar lipids (PLs) or rapeseed oil. There were four test meals (i) 15 g oat polar lipids: OPL, (ii) 18 g sunflower lecithin (of which 15 g were polar lipids): LPL, (iii) 18 g rapeseed oil: RSO, and (iv) reference white wheat bread: WWB. Lipid-enriched test meals contained equivalent amounts of total fat (18 g), and all breakfast meals contained 50 g available carbohydrates. The meals were served as breakfast followed by a standardised lunch (white wheat bread and meat balls) after 3.5 h. Test variables were measured at fasting and repeatedly during 5.5 h after ingestion of the breakfast.

**Results:**

Our study demonstrated that both LPL and OPL had beneficial effects on postprandial glucose and insulin responses, and appetite regulating gut hormones, as compared to RSO and WWB. Significant increase in GLP-1, GIP, and PYY concentrations were seen after consuming breakfast meals with LPL and OPL, and ghrelin concentration was reduced compared to meals with RSO and WWB (*p* < 0.05). Furthermore, triglycerides (TG) concentration was significantly reduced after OPL compared to RSO (*p* < 0.05). Our data show that there were no significant variations in glycaemic and insulin responses, TG, and gut hormone concentrations between LPL and OPL during breakfast (0–210 min) or over the whole study period (0–330 min).

**Conclusion:**

Our study revealed that the consumption of both lecithin and oat PLs included in breakfast meal may similarly enhance postprandial glucose tolerance, reduce TG, and enhance the secretion of incretins and appetite regulating hormones in healthy young adults.

**Clinical trial registration:**

ClinicalTrials.gov, identifier NCT05139355.

## Introduction

Over the last decades, there has been a concerning increase in the worldwide prevalence of lifestyle-related disorders such as obesity and type 2 diabetes mellitus (T2DM), which significantly increase the risk of cardiovascular diseases (CVD). Dietary habits are strongly involved in the pandemic prevalence, and probably also the most important modifiable factors that can be used in a preventive strategy. In this regard, intake of bioactive compounds found in whole grains, fruits and vegetables, nuts and oils are linked to a decreased likelihood of developing cardiometabolic diseases (CMD) (1–4).

Diet and food preferences may greatly influence the amount of bioactive polar lipids (PLs) included in the diet. Foods that consist of a higher amount of dietary PLs are nuts, egg, vegetable oils, dairy products, whole grains, fish and meats. The amount of dietary PLs widely varies depending on food choice and has been estimated that <1–10% PLs are included in total dietary lipids (1, 5). PLs, such as phospholipids and galactolipids, are major building blocks for biological cell membranes in almost all living species. Phospholipids, such as lecithin, are used food additives as natural emulsifiers (6, 7), and as antioxidants to reduce lipid oxidation (8). Biochemically, lecithin mainly refers to phosphatidylcholine (PC) but according to the official definition adopted by Food and Agriculture Organization of the United Nations (FAO) and European Food Safety Authority (EFSA), the term lecithin englobes different preparations containing at least 50% phospholipids (6, 9).

It has also been reported that lecithin exhibits various health promoting effects, including improved lipid digestion, anti-inflammatory activity and cholesterol lowering effects (7, 10–13). However, most of the research evaluating health effects of lecithin were done either *in vitro* or in animal models (14, 15). Thus, the nutritional impact of lecithin in humans remains to be explored.

Another interesting lipid rich in PLs are oat lipids. Recently, it has been shown that inclusion of oat PLs, rich in digalactosyldiacylglycerol (DGDG) and other galactolipids, in a breakfast meal improved postprandial and second-meal glycaemic tolerance, triglyceride concentrations and appetite-regulating gut hormones, in healthy humans (16, 17).

Oat PLs, despite their potential benefits, encounter more challenges in terms of production and cost-effectiveness compared to widely commercially used lecithins in food applications. Furthermore, the health benefits of oat PLs remain underexplored, and further studies are required to fully understand their impact and applications as a functional ingredient.

The purpose of this study was to investigate postprandial metabolic effects of sunflower lecithin incorporated in breakfast meals and compare them with those elicited by an equivalent amount of oat PLs. For this purpose, a randomized crossover meal study was performed in young healthy adults. Blood glucose, insulin, glucagon like peptide-1 (GLP-1), peptide tyrosine tyrosine (PYY), glucose dependent insulinotropic polypeptide (GIP), ghrelin and triglycerides (TG) were determined in the postprandial period following the test breakfast and after a subsequent standardised lunch.

## Materials and methods

### Study participants

Eighteen young healthy subjects; 11 male and 7 female, with a mean age of 25.6 ± 4.6 years, and BMI 23.6 ± 2.9 kg/m^2^ participated in the meal study. The inclusion criteria were age between 20 and 40 years and BMI between 19 and 28 kg/m^2^. Additionally, participants were required to be non-smokers and have no documented metabolic problems or food allergies. The intake of antibiotics or probiotics was prohibited for a period of 2 weeks before and during the trial period. Recruitments of test subjects took place between July to September 2021, and the clinical phase lasted from September to December 2021. Before being included in the study, every participant received a comprehensive explanation, both written and oral, of the objectives and methodology of the research. In addition, signed informed consent was collected from each participant. All participants were informed of their right to voluntarily withdraw from the trial at any point. The Consort flow diagram and study progress shown in Supplementary Figure S1.

### The test and standardised meals

A polar lipid-enriched oat oil (90% polar lipids) was specially prepared for the study and kindly provided by Swedish Oat Fiber AB (Bua, Sweden). Polar lipid-enriched (83% polar lipids) sunflower lecithin was purchased from Helhetshalsa AB (Borghamnsvägen 8, 59293, Borghamn, Sweden). These two polar lipids (PL) preparations were then used to prepare test meals. A white wheat bread (Jättefranska, Pågen AB, Sweden) was included in the breakfasts, as a source of available carbohydrates, both to be consumed with the lipids investigated, and as a reference product without added lipids. There were four meals tested in the study: The reference meal, the two different PL meals with equivalent amount of PL (15 g), and one oil with very low polar lipids meal [rapeseed oil (RSO)]. Prior to serving the test meals, polar lipids spreads were prepared by gently mixing oat oil or lecithin with 10 mL water. The spreads were then spread onto the WWB. Rapeseed oil was instead poured directly onto the WWB. The four test meals were noted as followed (i) 15 g oat polar lipids: OPL, (ii) 18 g sunflower lecithin (containing 15 g polar lipids): LPL, (iii) 18 g rapeseed oil: RSO, and (iv) reference white wheat bread: WWB. Lipid-enriched test meals contained equivalent amounts of total fat (18 g). The RSO meal was used as a common oil reference. All breakfast meals contained 50 g available carbohydrates and were consumed together with one glass of water (260 mL). Formulation and composition of the test meals are shown in Tables 1, 2.

**Table 1:** Formulation of the test and reference meals.

Reference meal^a^	WWB	120 g white wheat bread, no added oil, 260 mL water
Test meals^a^	OPL	120 g WWB, 16.6 g of polar lipids-rich (90%) oat oil (i.e., 15 g polar lipids) 1.4 g rapeseed oil, 260^b^ mL water.
	LPL	120 g WWB, 18.0 g of sunflower lecithin (i.e., 15 g polar lipids), and 260^b^ mL water.
	RSO	120 g WWB, 18 g rapeseed oil, 260 mL water.
Standardised lunch	120 g white wheat bread, 100 g meatballs, 250 mL water	

a WWB, white wheat bread containing 50 g available carbohydrates; OPL, 15 g oat polar lipids; LPL, 15 g lecithin; RSO, 18 g rapeseed oil.

b Including 10 mL water used for spread preparation.

**Table 2:** Macronutrient composition of breakfast.

	WWB	RSO	LPL	OPL
Carbohydrates (g)	50	50	50	50
Fats (g)	< 1	18	18	18
Polar lipids (g)*	< 1	0.6^*^	15	15
Non-Polar lipids (g)	< 1	17.4	3	3

Polar lipid contents in LPL and OPL according to the supplier and in ^*^RSO estimated according to reference (34).

The standardised lunch consisted of a meatball sandwich containing WWB, corresponding to 50 g available starch, and 100 g meatballs (Scan AB, Sweden). Water, 250 mL, was consumed to the meal. According to the nutritional information provided by the meatballs manufacturer, the lunch meal contained a total calorie value of 485 kcal (58 g of carbohydrates, 18.5 g of fat and 21.5 g of proteins).

### Study methodology and design

The study was carried out using a single-blind randomized crossover design. The participants paid 4 visits to the clinical unit. All participants consumed the 4 meals in a random order. Participants were given instructions to avoid intense physical activity, consuming alcoholic drinks, and taking foods that include oats or are high in dietary fiber (such as beans, whole grain bread, fiber-enriched pasta, and whole cereal kernels) before each study visit. The participants were directed to follow a consistent and uniform eating plan day before each trial day. To maintain uniformity, participants were requested to take note of their food consumption from the day before each study session. Furthermore, the participants were provided with clear instructions to eat a standardised dinner meal at 18:00 on the day before to each study visit. In addition, they were instructed to have an evening snack at 21:00, consisting of a commercial white wheat bread with a topping of their preference.

**Figure 1:**
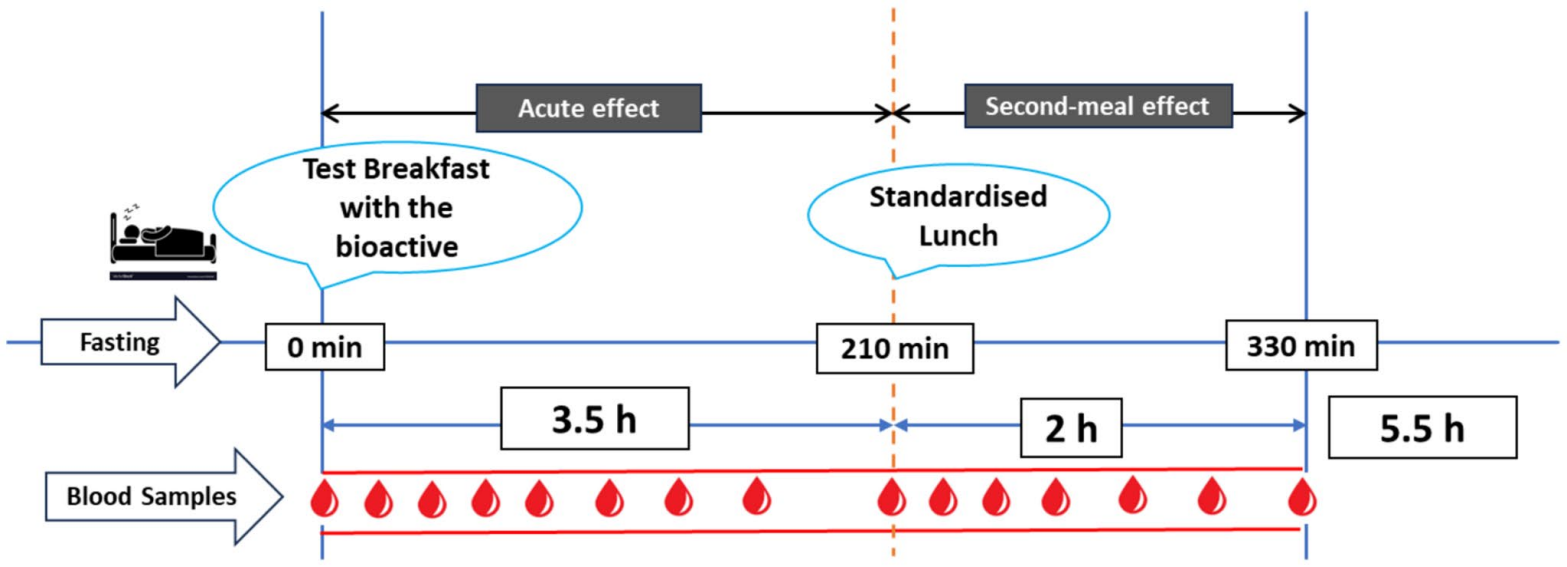
Overview of the clinical trial day.

An overview of the clinical trial day is presented in Figure 1. The test meals were given in the form of breakfast meals. Five-day interval was left between each test meal. The subjects arrived at the clinical unit at 07:30 following a 10-h period of fasting overnight. Fasting capillary blood samples were obtained., and then a test meal was consumed at time zero (0 min), with an even eating pace of 10–12 min. Consecutive capillary blood samples were obtained at time intervals of 15, 30, 45, 60, 90, 120, 150, and 210 min after the start of the breakfast meal. After taking a blood sample at the 210-min mark, a standardised lunch was given, and further blood samples were taken at intervals of 225, 240, 255, 270, 300, and 330 min after the beginning of breakfast. During the course of the experiment, the participants were restricted to the study facility and not allowed to consume any food or drinks, except from the breakfast and lunch meals that were served to them. They were also advised to reduce their physical activity as much as possible.

### Test variables

The measurement of all biomarkers in blood was carried out using capillary blood samples. Plasma glucose concentrations were measured in whole blood at the time intervals specified above, using a HemoCue Glucose 201+ analyzer (HemoCue AB, Ängelholm, Sweden). Samples for the analysis of serum insulin and TG were collected in BD Microtainer SST tubes. The serum insulin samples were taken at the same time points as the glucose determinations, except for the exclusion of 15 and 150 min. TG were measured at 0, 60, 120, 210, 270, and 330 min. The tubes were allowed to settle at the room temperature for 30 min prior to centrifugation for 10 min (2,000*g*) at a temperature of 25°C, using an Eppendorf centrifuge model 5,425. Subsequently, the serum was separated and kept at a temperature of −80°C until it was subjected to analysis.

Blood plasma samples were taken for the analysis of total concentrations of GLP-1, GIP, PYY, and ghrelin at four different time points (0, 60, 210, and 330 min). The samples were collected using BD Microtainer K2E tubes. A mixture of a DPP-4 inhibitor (10 μL/mL blood, Millipore, St. Charles, United States) and aprotinin (50 μL/mL blood, Sigma-Aldrich, St. Louis, USA) was injected into the tubes as GLP-1 inhibitory cocktail before the blood collections. The tubes were stored on ice prior to and during the collection of samples. Subsequently, the tubes were subjected to centrifugation for a duration of 10 min at 2,000*g* and a temperature of 4°C. This centrifugation process was carried out within a time frame of 30 min after the blood was collected. The plasma was thereafter separated, and samples were frozen at a temperature of −80°C until the time of analysis.

The measurement of insulin concentrations in blood serum was conducted using a solid phase two-site enzyme immunoassay kit (Insulin ELISA 10-1113-01, Mercordia AB, Uppsala, Sweden). The amounts of serum TG were measured using a multi-sample enzymatic assay known as LabAssay™ Triglyceride 290-63701, which follows the GPO.DAOS technique. This test was conducted by using analysis kit produced by FUJIFILM Wako Chemicals Europe GmbH, Germany. The measurement of total plasma concentrations of GLP-1, PYY, GIP, and ghrelin was conducted using a 10-spot U-plex test kit (Meso Scale Diagnostics LLC, Rockville, Maryland, United States). According to the supplier’s description, biotinylated capture antibodies are coupled to U-PLEX Linkers. The U-PLEX Linkers then self-assemble onto unique spots on the U-PLEX plate. After analytes in the sample bind to the capture reagents, detection antibodies conjugated with electrochemiluminescent labels (MSD GOLD SULFO-TAG) bind to the analytes to complete the sandwich immunoassay.

### Statistical calculations and data analysis

The data are presented as means ± SEM. The incremental areas and total areas under the curves (iAUC and AUC, respectively) were calculated by using a trapezoidal model for every participant and test meal. The iAUC was calculated for statistical analyses of blood glucose and insulin responses. AUC were used for assessing the TG, GLP-1, PYY, GIP, and ghrelin responses. The graph plotting and area calculation were conducted using GraphPad Prism (version 10.0.2, GraphPad Software, CA, United States).

The randomization of the consumption sequence of the test meals was achieved using the randomization features available in Microsoft Excel (Seattle, WA, USA). The variations in the results among different products (‘Meal’: OPL, LPL, RSO, and WWB) at different times throughout the experimental day (‘Time’) was assessed using a mixed model approach (PROC MIXED in SAS release 9.4; SAS Institute Inc., Cary, NC) with repeated measures and an autoregressive covariance structure for the test variables. The subjects were treated as a random variable, with the associated baseline (fasting values) being included as a covariate in the model. The physiological responses (AUC and iAUC) resulting from the test products were assessed using ANOVA (general linear model) followed by Tukey’s pairwise multiple comparison in MINITAB Statistical Software (version 21, Minitab, Minitab Inc., State College, PA, United States). The significance level was set at a *p*-value of less than 0.05.

### Power calculation

The primary outcome measure of the study was incremental blood glucose concentrations, iAUC, 0–120 min after the breakfast meal. The number of participants required for the study was determined based on a previous study (16). Assuming a difference of 22 mmol*min/L (10%) between test meals and a SD of 72 mmol*min/L, with *α* = 0.05 and 1 – *β* = 0.8 (16), 13 test subjects were required.

## Results

### Baseline characteristics

No statistically significant differences were seen in fasting concentrations of the reported variables. Data can be found in Table 3 (glucose and insulin), Table 4 (TG, GLP-1, PYY, GIP, and ghrelin).

**Table 3:** Glucose and insulin concentrations at fasting state and after intake of the test breakfast meals.

Test variables	WWB	RSO		LPL		OPL	
			%∆		%∆		%∆
Glucose							
Fasting blood glucose (mmol/L)	4.90 ± 0.13^a^	4.87 ± 0.12^a^	−0.57	4.73 ± 0.09^a^	−3.51	4.87 ± 0.09^a^	−0.57
Blood glucose prior to std. lunch (mmol/L)	4.65 ± 0.15^a^	4.70 ± 0.15^a^	1.08	4.89 ± 0.10^a^	5.26	5.02 ± 0.13^a^	8.00
Blood glucose iAUC 0–120 min (mmol*min/L)	181.3 ± 20.00^a^	145.10 ± 15.00^a^	−19.96	103.10 ± 12.70^b^	−43.13	104.40 ± 14.60^b^	−42.42
Blood glucose iAUC 210–330 min (mmol*min/L)	158.90 ± 12.20^a^	138.60 ± 13.90^ab^	−12.77	115.40 ± 8.16^bc^	−27.37	96.90 ± 11.10^c^	−39.01
Blood glucose iAUC 0–330 min (mmol*min/L)	324.40 ± 38.40^a^	297.50 ± 30.80^ab^	−8.29	221.20 ± 31.20^b^	−31.81	227.40 ± 27.80^b^	−29.90
Insulin							
Fasting blood insulin (nmol/L)	0.050 ± 0.004^a^	0.059 ± 0.005^a^	13.42	0.050 ± 0.004^a^	−4.83	0.053 ± 0.004^a^	1.91
Blood s-insulin prior to Std. lunch (nmol/L)	0.059 ± 0.011^a^	0.062 ± 0.012^a^	5.05	0.052 ± 0.009^a^	−11.12	0.059 ± 0.011^a^	0.50
Insulin iAUC 0–120 (nmol*min/L)	24.19 ± 2.87^a^	20.20 ± 2.05^a^	−16.49	13.76 ± 1.65^b^	−43.12	13.14 ± 1.91^b^	−45.68
Insulin iAUC 210–330 (nmol*min/L)	24.13 ± 3.21^a^	19.96 ± 2.53^a^	−17.28	12.47 ± 1.67^b^	−48.32	13.63 ± 2.03^b^	−43.51
Insulin iAUC 0–330 (nmol*min/L)	54.68 ± 6.53^a^	44.70 ± 4.61^a^	−18.25	29.16 ± 3.28^b^	−46.67	30.92 ± .60^b^	−43.45

Data are reported as means ± SEM, *n* = 18. Different superscript letters indicate statistically significant differences between values in the same row, *p* < 0.05 (ANOVA, followed by Tukey’s test). The percentage change is obtained as the difference from the WWB. WWB, white wheat bread; OPL, 15 g oat polar lipids; LPL, 15 g lecithin (polar lipids); RSO, 18 g rapeseed oil; iAUC, incremental area under curve.

**Table 4:** TG and gut hormones concentrations at fasting state and after intake of the test breakfast meals.

Test variables	WWB	RSO		LPL		OPL	
			%∆		%∆		%∆
TG							
Fasting TG (mmol/L)	0.63 ± 0.03^a^	0.69 ± 0.03^a^	10.1	0.60 ± 0.03^a^	−4.25	0.62 ± 0.03^a^	−1.39
TG AUC 0–210 min (mmol*min/L)	153.27 ± 8.49^a^	196 ± 13.70^b^	27.88	165.85 ± 8.95^a^	8.21	152.73 ± 8.34^a^	−0.35
TG AUC 210–330 min (mmol*min/L)	93.28 ± 5.00^a^	121.09 ± 7.67^b^	29.81	107.28 ± 7.18^ab^	15.01	99.73 ± 5.68^a^	6.91
TG AUC 0–330 min (mmol*min/L)	246.5 ± 13.10^a^	317.10 ± 19.90^b^	28.64	273.1 ± 15.40^a^	10.79	252.50 ± 13.4^a^	2.43
GLP-1							
Fasting plasma GLP-1 (pg/mL)	33.16 ± 1.83^a^	32.3 ± 1.79^a^	−2.59	33.32 ± 1.59^a^	0.48	36.96 ± 2.62^a^	11.45
GLP-1 AUC 0–210 (pg*min/mL)	8,007 ± 352^a^	9,438 ± 456^b^	17.87	12,034 ± 460^c^	50.29	13,187 ± 616^c^	64.69
GLP-1 AUC 0–330 (pg*min/mL)	12,689 ± 441^a^	14,280 ± 624^a^	12.54	18,248 ± 768^b^	43.8	20,553 ± 996^c^	61.97
GLP-1 at 330 min (pg/mL)	50.19 ± 2.79^a^	47.73 ± 2.61^a^	−4.90	61.11 ± 4.61^b^	21.75	71.41 ± 4.17^c^	42.27
PYY							
Fasting plasma PYY (pg/mL)	33.41 ± 2.26^a^	34.21 ± 1.99^a^	2.39	34.05 ± 2.00^a^	1.92	37.42 ± 2.53^a^	12.01
PYY AUC 0–210 (pg*min/mL)	7,737 ± 526^a^	8,395 ± 655^a^	8.50	10,981 ± 913^b^	41.93	11,934 ± 641^b^	54.24
PYY AUC 0–330 (pg*min/mL)	12,330 ± 788^a^	13,577 ± 1007^a^	10.11	18,633 ± 1295^b^	51.12	20,196 ± 1099^b^	63.79
PYY at 330 min (pg/mL)	43.56 ± 5.07^a^	47.97 ± 3.84^a^	10.12	75.74 ± 4.23^b^	73.88	81.57 ± 5.26^b^	87.25
GIP							
Fasting plasma GIP (pg/mL)	151.43 ± 6.23^a^	144.88 ± 4.75^a^	−4.33	140.91 ± 2.61^a^	−6.95	142.9 ± 3.01^a^	−5.63
GIP AUC 0–210 (pg*min/mL)	40,797 ± 2023^a^	77,834 ± 4832^b^	90.78	50,392 ± 2743^ac^	23.52	55,118 ± 2732^c^	35.11
GIP AUC 0–330 (pg*min/mL)	75,778 ± 4532^a^	115,885 ± 7970^b^	52.93	85,320 ± 4005^ac^	12.59	92,248 ± 5265^c^	21.73
GIP at 330 min (pg/mL)	439.80 ± 44.10^a^	431.9 ± 57.20^a^	−1.79	417.5 ± 41.20^a^	−5.07	451.90 ± 42.80^a^	2.75
Ghrelin							
Fasting plasma Ghrelin (pg/mL)	372.20 ± 19.90^a^	387.70 ± 24.30^a^	4.16	354.60 ± 20.10^a^	−4.72	341.00 ± 19.40^a^	−8.38
Ghrelin AUC 0–210 (pg*min/mL)	70,023 ± 5448^a^	71,240 ± 5208^a^	1.73	53,426 ± 2288^b^	−23.7	48,608 ± 2240^b^	−30.58
Ghrelin AUC 0–330 (pg*min/mL)	115,898 ± 9341^a^	114,515 ± 8517^a^	−1.19	84,065 ± 3633^b^	−27.46	76,028 ± 3317^b^	−34.4
Ghrelin at 330 min (pg/mL)	291.00 ± 33.30^a^	275.30 ± 31.70^a^	−5.39	181.10 ± 10.40^b^	−37.766	189.30 ± 18.70^b^	−34.94

Data are reported as means ± SEM, *n* = 18. Different superscript letters indicate statistically significant differences between values in the same row, *p* < 0.05 (ANOVA, followed by Tukey’s test). The percentage change is obtained as the difference from the WWB. WWB, white wheat bread; OPL, 15 g oat polar lipids; LPL, 15 g lecithin (polar lipids); RSO, 18 g rapeseed oil; AUC, area under curve.

### Glucose and insulin

**Figure 2:**
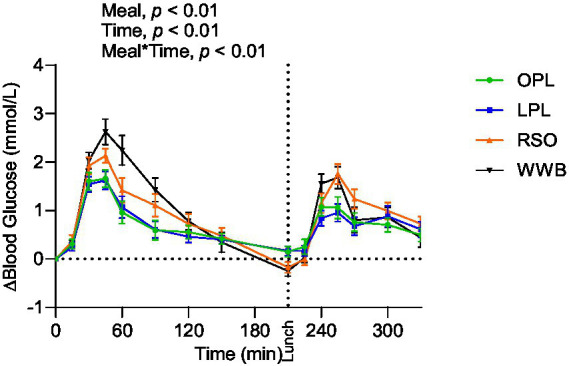
The incremental changes in blood glucose concentration after the consumption of breakfasts and standardised lunch meals. The values are expressed as means ± SEM. Repeated measures; mixed model in SAS. OPL, 15 g oat polar lipids; LPL, 15 g lecithin (polar lipids); RSO, 18 g rapeseed oil; WWB, white wheat bread.

The meals showed a significant main effect (0–330 min) on blood glucose responses, as well as meal*time interactions (Figure 2). The glucose iAUC (0–120 min) following the breakfast meal with OPL and LPL was significantly reduced compared to WWB (*p* < 0.001) and RSO (*p* < 0.01) (Table 3). Regarding the glycaemic response to the standardised lunch (iAUC 210–330 min), the OPL and LPL were significantly lower compared to WWB (*p* < 0.01). No significant differences in blood glucose concentrations (iAUC 210–330 min) were observed with LPL compared to RSO. However, blood glucose concentration (iAUC 210–330 min) was significantly decreased after consumption of OPL at breakfast compared to RSO (*p* < 0.01). In addition, the postprandial glucose concentrations along the entire experiment (iAUC 0–330 min) were lower following OPL and LPL compared to WWB (*p* < 0.05).

**Figure 3:**
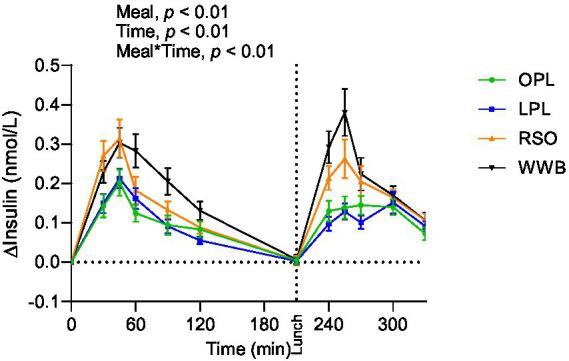
The incremental changes in blood insulin concentration after the consumption of breakfasts and standardised lunch meals. The values are expressed as means ± SEM. Repeated measures; mixed model in SAS. OPL, 15 g oat polar lipids; LPL, 15 g lecithin (polar lipids); RSO, 18 g rapeseed oil; WWB, white wheat bread.

A main effect of meal was observed on insulin responses during the whole experimental period (0–330 min, Figure 3), showing significantly lower postprandial insulin responses (iAUC 0–120 min) after breakfast following OPL and LPL compared to the breakfasts with WWB (*p* < 0.001) and RSO (*p* < 0.01). Furthermore, the insulin responses to the standardised lunch (iAUC, 210–330 min), were significantly lower after consuming OPL and LPL breakfasts compared to WWB and RSO (*p* < 0.05, Table 3). Thus, the postprandial insulin concentrations during the entire experimental session (0–330 min) were significantly lower after OPL and LPL compared to WWB and RSO (*p* < 0.01).

### Triglycerides

**Figure 4:**
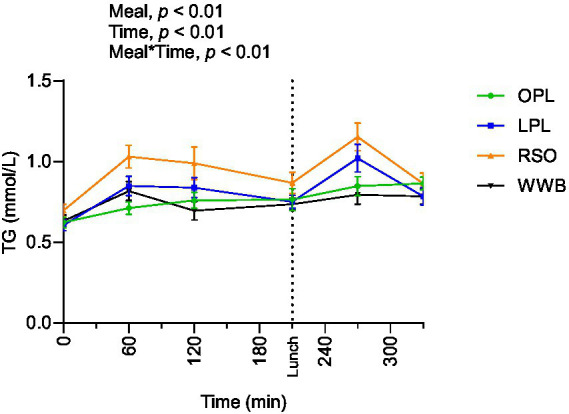
The changes in TG concentration after the consumption of breakfasts and standardised lunch meals. The values are expressed as means ± SEM. Repeated measures; mixed model in SAS. OPL, 15 g oat polar lipids; LPL, 15 g lecithin (polar lipids); RSO, 18 g rapeseed oil; WWB, white wheat bread.

Significant main effect of meals on TG concentration were found along the entire test period (0–330 min, Figure 4). The TG responses after breakfast (AUC 0–210 min) and lunch (210–330 min) were significantly lower after intake of OPL and LPL compared to after RSO (*p* < 0.05, Table 4). As expected, the WWB breakfast resulted in the lowest concentrations of TG during the test period, however no significant differences were detected in TG concentrations during the experimental period after intake of WWB compared with OPL and LPL (*p* > 0.05).

### GLP-1

**Figure 5:**
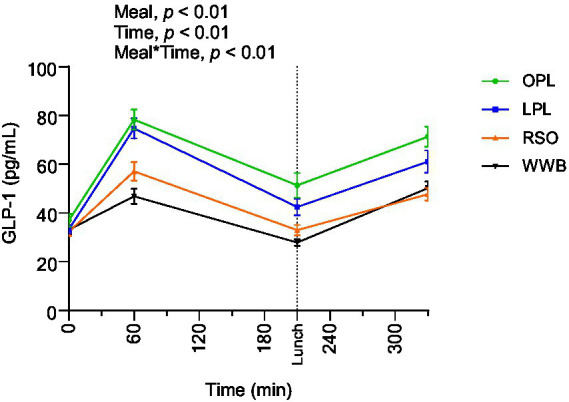
The changes in GLP-1 concentration after the consumption of breakfasts and standardised lunch meals. The values are expressed as means ± SEM. Repeated measures; mixed model in SAS. OPL, 15 g oat polar lipids; LPL, 15 g lecithin (polar lipids); RSO, 18 g rapeseed oil; WWB, white wheat bread.

The GLP-1 responses are presented in Figure 5 and Table 4. A significant main effect of meals and meal*time was detected along the entire test period (0–330 min, *p* < 0.01). The GLP-1 concentrations (AUC) during the time period 0–210 min were significantly increased after breakfast containing OPL and LPL compared to WWB and RSO (*p* < 0.05). Additionally, GLP-1 concentrations during 0–330 min were significantly higher after OPL and LPL breakfast compared to WWB and RSO breakfasts (*p* < 0.05).

### PYY

**Figure 6:**
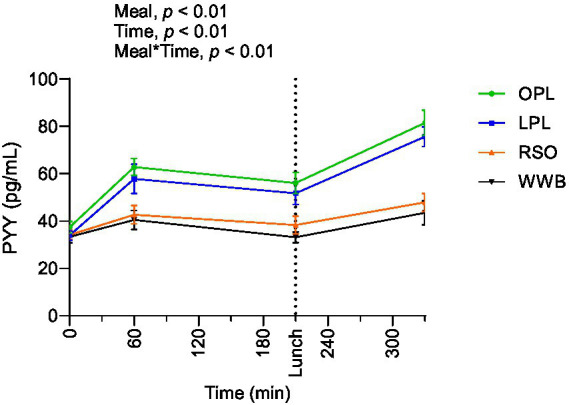
The changes in PYY concentration after the consumption of breakfasts and standardised lunch meals. The values are expressed as means ± SEM. Repeated measures; mixed model in SAS. OPL, 15 g oat polar lipids; LPL, 15 g lecithin (polar lipids); RSO, 18 g rapeseed oil; WWB, white wheat bread.

Results of PYY concentrations are shown in Figure 6 and summarized in Table 4. A significant main effect of meal and a significant meal*time interaction was observed along the test period (0–330 min, *p* < 0.05). The PYY concentrations during the whole investigation period (AUC 0–330 min) were significantly higher after OPL and LPL breakfast compared to WWB (*p* < 0.05) and RSO (*p* < 0.05).

### GIP

**Figure 7:**
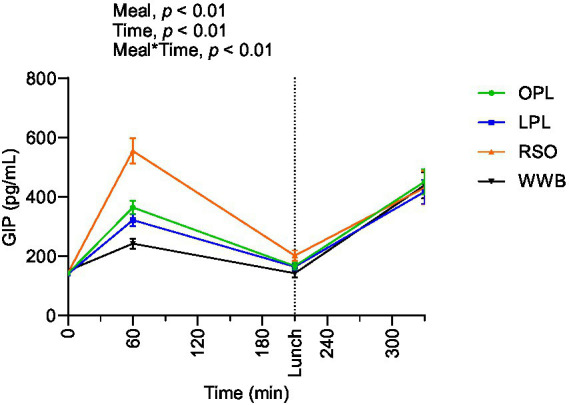
The changes in GIP concentration after the consumption of breakfasts and standardised lunch meals. The values are expressed as means ± SEM. Repeated measures; mixed model in SAS. OPL, 15 g oat polar lipids; LPL, 15 g lecithin (polar lipids); RSO, 18 g rapeseed oil; WWB, white wheat bread.

The postprandial outcomes of GIP after the consumption of breakfast and standardised lunch meals are shown in Figure 7 and summarized in Table 4. Significant main effects of the meal and meal*time on GIP concentrations were observed over the study period 0–330 min. The GIP concentrations (AUC) during the 0–210 min time period were significantly lower after breakfast containing OPL and LPL compared to RSO (*p* < 0.05), and the significant differences were detected also when investigating the entire experimental period (AUC 0–330 min, *p* < 0.05). In addition, GIP concentration during 0–330 min was significantly lower after OPL and LPL breakfast compared to RSO breakfasts (*p* < 0.05).

### Ghrelin

**Figure 8:**
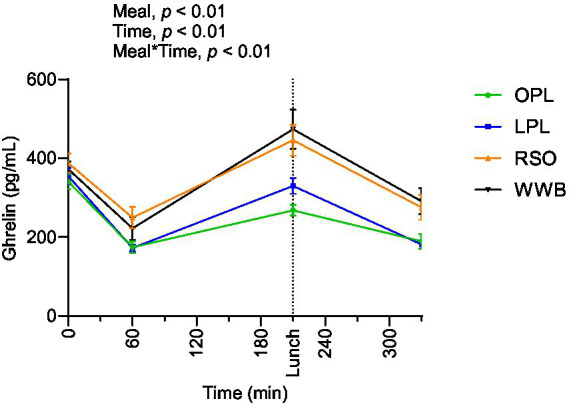
The changes in ghrelin concentration after the consumption of breakfasts and standardised lunch meals. The values are expressed as means ± SEM. Repeated measures; mixed model in SAS. OPL, 15 g oat polar lipids; LPL, 15 g lecithin (polar lipids); RSO, 18 g rapeseed oil; WWB, white wheat bread.

The results showed significant main effect of meal and a meal*time interaction over the test period 0–330 min (Figure 8). Postprandial ghrelin concentrations (AUC 0–330 min) were significantly lower after OPL and LPL breakfasts compared to RSO and WWB (*p* < 0.05, Table 4).

## Discussion

The objective of this research was to investigate the metabolic effects of 15 g polar lipids in a sunflower lecithin preparation on glucose tolerance, TG concentrations, and gut hormones (GLP-1, GIP, PYY, and ghrelin) in young healthy individuals, and compare them with the effects of an equivalent amount of OPL. The metabolic variables were investigated postprandially after breakfast and a subsequent lunch. The results demonstrated that both LPL and OPL elicited beneficial effects on postprandial glucose and insulin responses, in comparison to RSO and WWB. Furthermore, a significant increase was observed in concentrations of GLP-1, GIP, PYY, whereas the concentration of ghrelin was suppressed after the breakfast meals with LPL and OPL compared to those with RSO and WWB. Importantly, our results indicate no significant differences in glycaemic and insulin responses, TG and gut hormones concentrations between LPL and OPL after breakfast (0–210 min), or when investigating the whole experimental period (0–330 min).

The results in the present study is in accordance with previous investigations in our research group, demonstrating that 12 g (17) and 15 g (16) OPL have the potential to reduce postprandial glucose and insulin responses, lower concentrations of TG and ghrelin, and enhance the release of GLP-1 and PYY. Taken together, the results in the presently described study indicate that LPL and OPL may elicit similar health impact by modulating postprandial cardiometabolic variables.

It has been shown that incorporation of fat in carbohydrate rich meals in general reduce the glycaemic responses (18). However, the current study demonstrates that LPL and OPL exert effects on postprandial glucose tolerance beyond the established knowledge regarding the impact of fat on postprandial glycaemic response. Consequently, the glucose responses after LPL and OPL were more importantly reduced as compared with similar amount of RSO. In addition, both breakfast meals with LPL and OPL significantly decreased glucose and insulin responses during whole investigated period (iAUC 0–330 min) compared to WWB, whereas no such effects were found after consumption of RSO and WWB. The prolonged benefits on glucose tolerance observed across two consecutive postprandial periods enhance the antidiabetic potential of a food or functional ingredient, beyond what is achieved by foods simply classified as having a low glycaemic index. Additionally, our study shows that LPL and OPL also reduced insulin responses, not only acutely after intake (iAUC 0–210 min) but also after the following lunch (iAUC 210–330 min) compared to RSO and WWB. No reduction in postprandial or second meal insulin responses was observed after RSO when compared to WWB.

Previous research has revealed that ingestion of a fat to carbohydrate rich meal slows gastric emptying rate, promote secretion of incretin hormones (GLP-1 and GIP) and modulate other appetite regulating hormones such as PYY and ghrelin (19–22). Studies conducted in T2DM patients with olive oil (containing only minor amount of polar lipids) (23) demonstrated that consuming fat 30 min prior to a meal rich in carbohydrates has a significant effect on the rate of gastric emptying, which leads to delayed fat digestion and thus contribute to improve postprandial glycaemic excursion. The results in our study do not support effects on gastrointestinal hormones in healthy humans of the RSO. Such effects, however, were observed after LPL and OPL. It can be suggested that the underlying mechanisms to the here-observed effects on glucose regulation of LPL and OPL are at least partly due to a reduced gastric emptying rate and delayed and/or decreased fat digestion. A delayed fat absorption could potentially enhance the release of gut hormones (23).

The currently described study indicate lowering effects on circulating TG of lecithin from sunflower and a PL-rich oat oil preparation compared with a conventional oil (RSO), in an acute meal setting (AUC 0–330 min). Such a TG reducing effect of sunflower lecithin is in line with previous observations with long term ingestion of soybean lecithin (10). A reduction in plasma TG concentrations was also observed in hypertriglyceridemic patients consuming 12 g of soya lecithin daily for 3 months (14, 15).

The LPL preparation used here was naturally rich in phospholipids (83% PL), containing a mixture of PC, phosphatidylethanolamine (PE), phosphatidylinositol, phosphatidylserine, and phosphatidic acid, whereas the OPL preparation was rich in galactolipids (mainly DGDG), but also contained significant amounts of naturally occurring phospholipids.

A stable phospholipid composition of hepatocytes, especially the ratio of PC/PE, is essential in insulin signaling (24) and in regulating glucose and energy metabolism in the liver (25). Abnormal PC/PE ratios influence energy metabolism and are associated with liver diseases (26). Dietary supplementation with vegetable lecithins and oat PLs, particularly because of their PC and PE fractions, may contribute to balance this PL ratio in the liver and hence improve insulin sensitivity and glucose regulation.

Oat oil contains relatively high amounts (3.5 pmol/mg) of branched fatty acid esters of hydroxy fatty acids (estolides) (27–29). To our knowledge no data has been reported regarding naturally occurring estolides in sunflower but their presence in LPL cannot be ruled out. Estolides have drawn attention due to their potential effects on metabolic health. Some reports indicate that estolides may exert anti-diabetic and anti-inflammatory effects, and are suggested as a potential nutraceutical bioactive lipids (30–33). Thus, in addition to what was mentioned above, another mechanism behind the beneficial metabolic effects of LPL and/or OPL could be linked to the presence of naturally occurring estolides in these PLs. Ohlsson et al., reported that fractionated oat oil liposomes containing PL, which naturally contains a high proportion of DGDG estolides, delayed postprandial lipid digestion, reduced total energy intake, and modified satiety and appetite in healthy humans (30). Yore M. et al., reported that estolids have the potential to improve glucose tolerance and stimulate GLP-1 and insulin secretion both in humans and mice (33). Additionally, 12-week supplementation of estolides (0.37 mg per day) resulted in improved insulin sensitivity in a mouse model (31). Further studies exploring the mechanisms governing the beneficial impact of dietary PL on metabolic health are needed.

A limitation of the study related to the sample size, which was determined based on the primary endpoint of the study, i.e., postprandial change in blood glucose concentrations. However, this may result in insufficient statistical power for the secondary metabolic markers investigated. This study was conducted as a single-blinded trial due to the nature of the test meals. The differences in appearance, texture, and taste between the meals with the lipid preparations tested (lecithin, oat polar lipids and rapeseed oil) and the white wheat bread reference meal ruled out a double-blinding strategy. However, to minimize bias, the participants were blinded to the type of lipid being consumed. The researchers involved in the meal preparation and distribution were aware of the meal composition, but those responsible for collecting and analysing the postprandial blood samples and conducting data analysis were blinded to the treatment conditions. Additionally, we did not measure the amounts of estolides in the test meals, nor in the blood samples. In the context of elucidating the mechanisms through which lecithin and OPL exert their metabolic effects, we have hypothesized that their shared amphipathic properties underlie a common mode of action. It is important to acknowledge the possible limitations of this hypothesis due to the structural differences among the major components of these lipid preparations such as phospholipids and galactolipids. The differences in structure between the two lipid preparations not only differentiate them at a molecular level but also may have different response mechanisms and metabolic functions. Assuming that all polar lipids behave uniformly because of their amphipathic properties may be an oversimplification. The unique structural features of galactolipids and phospholipids suggest that they may have distinct effects on the signaling pathways and physiological responses.

Despite its limitations, this study has several notable strengths. First, the crossover design allowed each participant to serve as his/her own control, reducing inter-individual variability thus increasing the statistical power of the trial. Second, the study measured several metabolic markers, such as postprandial glucose, insulin, and triglycerides, as well as appetite-regulating gut hormones (GLP-1, GIP, PYY, and ghrelin). This provided an encompassing view of the effects of lecithin and oat polar lipids on metabolic health. Additionally, the use of real-world meal conditions, with a food commonly consumed in daily life, enhances the validity and practical relevance of the findings.

## Conclusion

In this randomized crossover study, we demonstrated that the inclusion of oat polar lipids and sunflower lecithin in a meal can similarly enhance postprandial glucose tolerance, reduce triglyceride levels, and promote the secretion of incretins and appetite-regulating hormones in healthy young adults. The significant increases in GLP-1 and PYY, along with reduced ghrelin concentrations, suggest that these bioactive lipids may help in regulating appetite and improving postprandial metabolic responses. Given the increasing prevalence of metabolic disorders, such as obesity and T2DM, the results highlight the potential use of these lipids as part of dietary interventions to improve postprandial metabolic health. Nevertheless, follow-up studies should focus on investigating the long-term metabolic effects of PL preparations, including dose–response evaluation and the mechanism(s) governing their metabolic effects.

## Data Availability

The raw data supporting the conclusions of this article will be made available by the authors, without undue reservation.
