# Supplementary figures and images for: Oat polar lipids and sunflower lecithin similarly improve cardiometabolic risk markers and appetite controlling hormone responses after breakfast and a subsequent lunch. A randomized crossover study in healthy adults
Source: Front Nutr. 2024 Nov 6;11:1497844. doi: 10.3389/fnut.2024.1497844 (PMC11576272; doi:10.3389/fnut.2024.1497844)

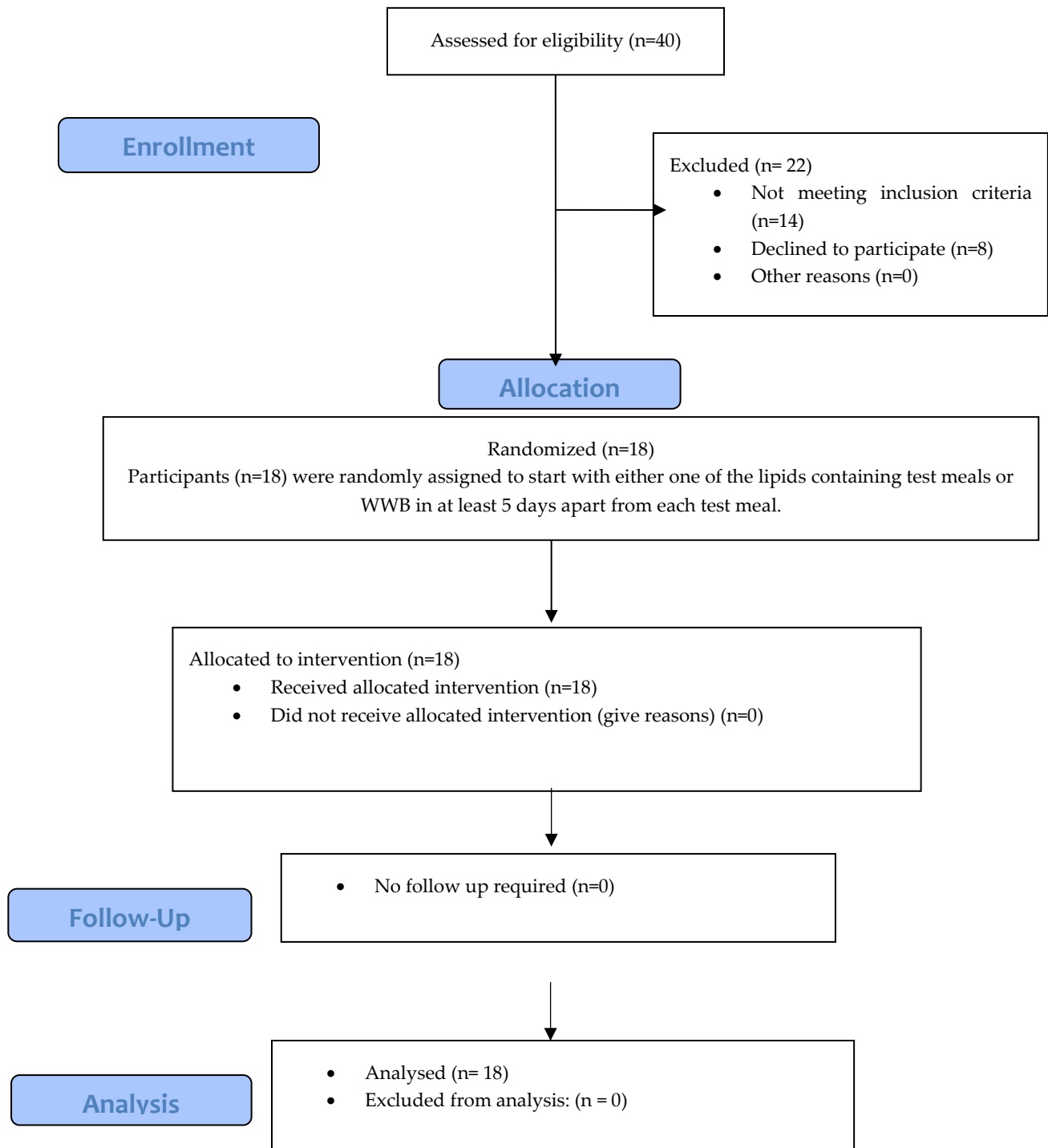

Figure I: Consort flow diagram of the study progress

Supplement: Supplementary file 1 [file Image_1.pdf]
